# The Association Between Anesthetist Leadership Styles and Their Resilience: A Single-Center Study

**DOI:** 10.7759/cureus.80848

**Published:** 2025-03-19

**Authors:** Laurence Weinberg, Elizabeth P Hu, Mark M Youssef, Benjamin Churilov, Dong-Kyu Lee

**Affiliations:** 1 Department of Anesthesia, Austin Health, Melbourne, AUS; 2 Department of Critical Care, The University of Melbourne, Parkville, AUS; 3 Department of Anesthesiology and Pain Medicine, Dongguk University Ilsan Hospital, Goyang, PRK

**Keywords:** anesthesia, authentic leadership, leadership, resilience, transformational leadership, workplace resilience

## Abstract

Purpose: To explore the association between the leadership styles and resilience of anesthetists in leadership roles.

Methodology: This prospective study of all anesthetists in leadership roles within a university teaching hospital in Melbourne, Australia, employed three validated, anonymous questionnaires (Multifactor Leadership Questionnaire, Authentic Leadership Questionnaire, and the Resilience Scale). Questionnaire data were summarized using descriptive statistics and presented as counts and proportions. Spearman’s rank correlation was used to evaluate quantitative continuous variables to identify linear relationships.

Results: Of the 14 anesthetists in leadership roles, 14 completed all three questionnaires (response rate 100%). Ten participants (71.5%) displayed behaviors consistent with transformational leadership. Three participants (21.4%) displayed behaviors typically associated with transactional leadership, and one (7.1%) exhibited characteristics consistent with passive-avoidant leadership. Anesthetists with transformational leadership styles exhibited significantly higher resilience scores than those with transactional or passive-avoidant leadership styles. Those with transformational leadership qualities also had increased job satisfaction overall.

Conclusion: Anesthetists who exhibit transformational and authentic leadership are likely to be more resilient than those with transactional or passive-avoidant leadership styles. Higher levels of resilience were also associated with greater role effectiveness and experiencing greater levels of job satisfaction.

## Introduction

The link between leadership styles and resilience is under-researched. Leadership within the medical profession plays a pivotal role in shaping the resilience of physicians who frequently perform their duties under stressful conditions and in demanding environments. Strategies that healthcare leaders could use to cultivate resilience in medical teams have emerged as a topic of growing significance. While leadership and/or resilience have been explored in a variety of healthcare settings [1-3], to date, no research has been undertaken on the association between anesthetists’ leadership styles and their resilience as leaders.

Transformational leadership is currently a topic of significant interest and concern among those seeking to improve the practice of medicine and healthcare in general [4]. Transformational leaders motivate their teams by supporting a shared vision and providing intellectual stimulation, individualized consideration, and inspirational motivation [5]. Within healthcare, transformational leaders create environments that support, value, and encourage excellence among physicians and healthcare teams. The results from several recent studies reveal that healthcare providers supported by transformational leaders frequently exhibit higher levels of resilience [6]. Transformational leaders provide emotional support and foster open communication styles that provide physicians with the capacity to cope with the daily pressures and challenges inherent in their professional work [4].

By contrast, autocratic or transactional leadership focuses on structured rules, clear expectations, hierarchies, and rewards or punishments for performance. While these leadership styles may ensure strict adherence to protocols, they may not promote physician resilience and may contribute to feelings of burnout and decreased job satisfaction [7,8]. Physicians working with autocratic or transactional leaders typically experience reduced autonomy and can find it challenging to adapt to the dynamic and often unpredictable nature of the healthcare environment. Thus, these two leadership styles may be minimally effective at promoting physician resilience. Furthermore, the autocratic or “authoritarian” leadership style leads to burnout and decreased job satisfaction and ultimately undermines the capacity of physicians to recover from adversity [8]. Several scholars have emphasized the importance of collaborative and empowering leadership styles, particularly in academic hospital settings [9]. Leaders who involve physicians in decision-making and provide opportunities for skill development can enhance physician resilience by fostering a sense of autonomy and control [10].

Due to the complex nature of their work, anesthetists in leadership roles face a multitude of stressors, notably the need to integrate academic and administrative responsibilities with ongoing care of high-risk clinical cases. The correlation between different leadership styles and the personal resilience of anesthetists in the context of leadership positions has not been explored previously.

To address this, we performed a prospective study to explore the association between leadership styles and resilience exhibited by anesthetists in leadership roles.

This study focused exclusively on anesthetists in leadership roles to examine the relationship between leadership styles and resilience within a defined group responsible for guiding and managing teams. By targeting anesthetist leaders, we sought to gain insights into how leadership behaviors influence resilience in high-pressure clinical environments, where effective decision-making, team dynamics, and role modeling are essential. Including all anesthetists within the department would introduce variability in leadership experience and responsibilities, potentially diluting the findings and limiting the ability to draw meaningful conclusions about leadership-specific traits and their impact on resilience.

## Materials and methods

Setting and design

This study was conducted by Austin Health, a 900-bed university teaching hospital in Melbourne, Australia. The hospital is the only center within Victoria that provides services for acute spinal injuries, liver transplantation, and toxicology. The Department of Anesthesia at Austin Health has 132 consultant anesthetists who perform services for >45,000 elective and emergency procedures across two campuses.

The inclusion criteria were anesthetists within the department of anesthesia who served in leadership roles. These roles included members of the anesthesia executive, the deputy directors, and the anesthesia heads of research, perioperative medicine, cardiac anesthesia, simulation, and pain medicine. The anesthesia executive at Austin Health provides overarching leadership, aligning the department's goals with organizational strategy, ensuring optimal resource allocation, and fostering a culture of excellence. Deputy directors play pivotal roles in managing critical domains, such as staffing, internal operations, and governance, ensuring smooth functioning and equitable support for all team members. Heads of research, perioperative medicine, cardiac anesthesia, simulation, and pain medicine contribute specialized expertise, driving innovation, evidence-based practice, multidisciplinary collaboration, and education while addressing specific patient care and training priorities. Together, they form a cohesive leadership team that ensures comprehensive, forward-thinking decision-making for the department. Exclusion criteria included clinical anesthetists in non-leadership positions, anesthesia nurse unit managers, and non-medical anesthesia business unit managers.

After obtaining approval from the Austin Health Research Ethics Committee (approval number: HREC/110843/Austin-2024), participants were invited by email to participate in the study (see Supplementary File 3, Table 2). Participants were provided with an information form that outlined the purposes of the research (see Supplementary File 3, Table 3). By choosing to complete and submit the survey, participants indicated their voluntary agreement to participate. The information form also stated that, if participants chose to complete the data collection and surveys and submit it for analysis, this would be evidence of their implied consent. The information form provided details about the study's purpose, procedures, potential risks, and benefits, ensuring that participants had sufficient information to make an informed decision. No personal identifiers were collected, and responses were anonymous to maintain confidentiality. Participants were assured that they could withdraw from the survey at any time without penalty, ensuring their autonomy and the voluntary nature of their participation in the study. All participant surveys were completed between 10 September 2024 and 17 September 2024. All surveys were anonymous.

Questionnaire surveys 

Three validated questionnaires were used to explore leadership styles and their relationship to the leaders’ resilience. Permission and licensing were obtained to comply with legal requirements and copyright regarding the use of these materials.

The Multifactor Leadership Questionnaire (MLQ)

The MLQ is a psychological assessment tool used to evaluate the entire spectrum of leadership styles [11]. It consists of 36 items focusing on leadership styles and nine items focusing on leadership outcomes (see Supplementary File 3, Table 2). In the questionnaire, five scales assess transformational leadership, two assess transactional leadership, and two assess passive and/or avoidant behavior. The MLQ also assesses effort, efficacy, and contentment or satisfaction as three dimensions of leadership outcomes. It has been validated in multiple cultural contexts and organizational settings. Over the past 25 years, it has served as the primary method for accurately distinguishing between highly effective and ineffective leaders in the military, government, education, manufacturing, technology, religion, hospitals, volunteer organizations, and other settings [9,10,12].

The Authentic Leadership Questionnaire (ALQ)

The ALQ, developed in 2007 (see Supplementary File, Table 3), is a sixteen-item tool used to assess authentic leadership behaviors [13]. This questionnaire is a self-assessment of self-awareness, internalized moral perspectives, balanced processing, and relational transparency, rather than authentic leadership as assessed by those being led. The items are used to generate four ALQ scales as follows: (i) the transparency scale reflects the extent to which a leader demonstrates openness and honesty in their interactions with others; (ii) the ethical/moral scale measures a leader’s moral principles and conduct; (iii) the balanced processing scale evaluates a leader’s ability to engage in fair and objective interpretation and decision-making by considering various perspectives; and (iv) the self-awareness scale measures a leader's capacity to recognize their own abilities, shortcomings, limitations, and how they are perceived by others.

The theoretical foundations of and empirical evidence underlying the concepts of transformational and authentic leadership are closely linked. Authentic leadership has been described as a basic concept that serves as the cornerstone of transformational leadership [14]. While transformational leadership emphasizes the connection of employees' sense of identity and self-worth to the organization, as well as their leadership capabilities, authentic leadership primarily focuses on efforts to nurture followers’ overall sense of self [15]. Moreover, both transformational and authentic leaders prioritize authentic acts and behaviors [16]. Both also highlight the significance of self-awareness, positive role modeling, encouraging follower autonomy, positive interactions between followers and leaders, and the creation of an ethical and supportive organizational environment [9]. These similarities and relationships also provide supplementary leadership frameworks.

For the present study, ALQ scores were calculated for each ALQ scale. The sum of all responses for each scale item was calculated and divided by the total number of scale items. A score of 1-15 signifies weaker authentic leadership, while scores within 16-20 signify stronger authentic leadership. By comparing scores in each of these components, we ascertained the relative strengths and weaknesses of each participant in the research.

The ALQ has been validated by multiple studies performed across a range of cultural and work environments, based on original work reported by Walumbwa et al. [17]. We integrated ALQ responses with the MLQ to evaluate self-awareness, transparency, ethics/morality, and processing abilities exhibited by the study participants and thus measured the complete spectrum of authentic and transformational leadership behaviors.

The Resilience Scale

The resilience scale is a validated 25-item questionnaire that measures resilience in the work environment (see Supplementary File 3, Table 4) [18]. The scale consists of six levels of resilience, with a score of 25-100 signifying very low resilience, 101-115 low resilience, 116-130 moderately low resilience, 131-145 moderate resilience, 146-160 moderately high resilience, and 161-175 high resilience. It identifies five essential characteristics of resilience. First, self-reliance reflects belief in one's own talents and one’s capacity to rely on oneself to recognize personal positive attributes, strengths, and limitations [19,20]. Second, purpose indicates one’s recognition of a meaningful purpose in life and an appreciation of one's own contributions [21]. Third, equity implies that one can maintain a measured and well-balanced view of one's life and experiences and the capacity to regulate strong reactions to adversity [22,23]. Perseverance refers to persistence when confronted with hardship or discouragement. Finally, authenticity is the recognition that one’s journey is distinct and is accompanied by a sense of liberation and perception of one’s own distinctiveness [24].

Statistical analysis

The results of the questionnaires were summarized using descriptive statistics, with data presented as counts and proportions. Non-normal distributions were summarized and presented as medians and interquartile ranges. Because the sample size was small, all data were considered non-normally distributed. Spearman’s rank correlation was computed to assess the relationship between resilience and leadership styles. Spearman’s coefficient (rho) was presented to indicate the strength and direction of the relationship. The p-value and the 95% confidence interval are presented to provide a comprehensive understanding of the relationship between the variables and the precision of the estimate. The coefficient ranges from -1 to +1. A positive value indicates a positive monotonic relationship, while a negative value indicates a negative monotonic relationship. The closer the coefficient is to ±1, the stronger the monotonic relationship. The strength of the relationship was determined as follows: rho = 1: Perfect monotonic relationship; 0.8 ≤ rho < 1: Very strong monotonic relationship; 0.6 ≤ rho < 0.8: Strong monotonic relationship; 0.4 ≤ rho < 0.6: Moderate monotonic relationship; 0.2 ≤ rho < 0.4: Weak monotonic relationship; 0 < rho < 0.2: Very weak monotonic relationship; and rho = 0: No monotonic relationship.

## Results

Baseline characteristics

Of the 14 participants in leadership roles who were invited to participate, 14 (100%) completed all three questionnaires. The median age, duration of qualification (years since completion of anesthesia specialty training), formal leadership training or postgraduate qualifications in leadership, and number of years in a leadership position are summarized in Table 1.

**Table 1:** Baseline characteristics Data presented as number (proportions) and median (interquartile range).

Variable	Number of participants: 14
Age (years)	44 (40:57)
Male gender	11 (78%)
Female gender	3 (22%)
Duration of practice as an anaesthetist (years)	11 (9:24)
Duration in a leadership position (years)	3.5 (2:5.3)
Participants with formal postgraduate leadership qualifications	3 (21.4%)

Transformational leadership style

**Figure 1:**
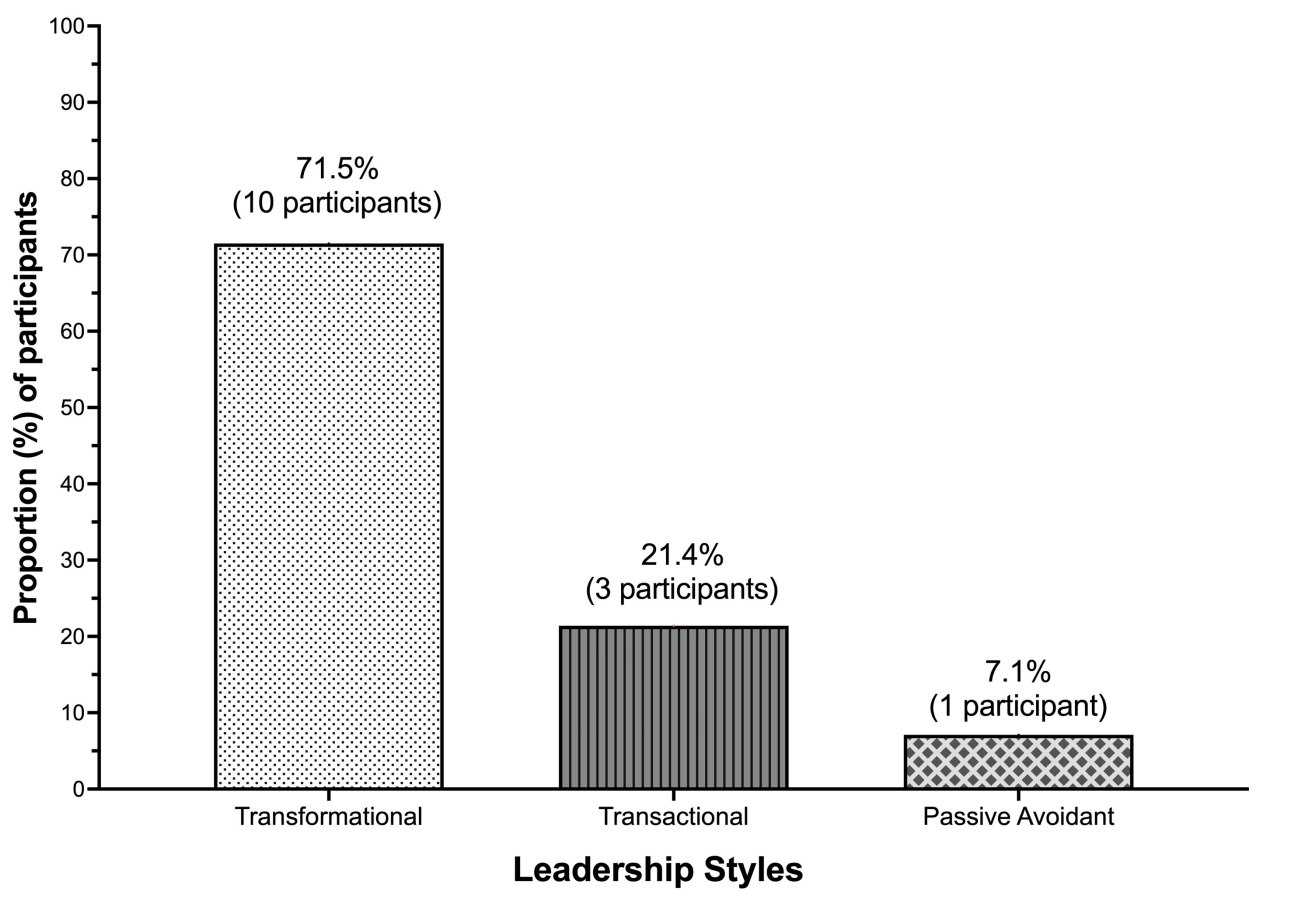
Bar graph showing leadership styles among anesthetists in leadership positions

In total, 10 participants (71.5%) reported behaviors consistent with transformational leadership (Figure 1). These leaders reported routinely fostering a sense of pride among their affiliated colleagues and acting selflessly on behalf of the department. They asserted that they conducted themselves in a manner intended to cultivate the admiration of others and demonstrated a sense of authority and self-assurance.

In response to the MLQ, participants with transformational leadership traits reported discussing their paramount values and convictions with others, the significance of maintaining a robust sense of purpose, and the need to foster a shared sense of mission. These leaders reported that they scrutinized their fundamental assumptions for ongoing suitability as leaders and sought diverse viewpoints when resolving issues. They reported frequently soliciting input from other department members, considering the impact of challenges from several perspectives, and proposing novel approaches to complete tasks. These anesthetists reported dedicating significant amounts of time to instruction and mentoring and regarding others as unique individuals rather than as group members. They acknowledged the unique needs, abilities, and goals of each individual, and stated that they were willing to assist others in cultivating their strengths.

Transactional leadership style

Three participants (21.4%) reported behaviors typically associated with transactional leadership (Figure 1). Two distinct transactional styles were apparent.

Anesthetists who followed the first style reported a constructive approach focused on rewarding individuals for their accomplishments. They also reported making their expectations clear, providing recognition when goals were met, establishing clear objectives, encouraging high levels of performance, offering incentives for successful outcomes, and closely monitoring others for any deviations or errors. In contrast to those reporting transformational leadership styles, this group indicated that they offered support to others in return for their efforts, clearly assigned responsibility for attaining performance goals, and provided clarity on rewards available to those who achieved stated objectives. They also reported exhibiting contentment when others fulfilled their expectations.

Anesthetists who followed the second style reported a focus on the continual monitoring of deviations and mistakes. They described having explicit guidelines for adherence and defining what qualifies as inadequate performance. At the same time, they asserted their authority to penalize those who failed to meet these standards. This leadership style involves vigilant oversight of deviations, mistakes, and errors, followed by prompt remedial measures designed to address irregularities, exceptions, and departures from established norms. A review of the MLQ responses revealed that these anesthetists reported focusing exclusively on addressing errors, grievances, and shortcomings, meticulously documenting all mistakes, and prioritizing the resolution of failures to meet established criteria.

Passivity and avoidance leadership style

One anesthetist (7.1%) reported characteristics consistent with “passive-avoidant behavior” (Figure 1). This participant reported a failure to provide systematic and timely responses to circumstances and crises and actively avoided the establishment of clearly defined agreements, expectations, goals, and standards. Because this participant tended to refrain from engaging with others, this leadership style might be more accurately described as “non-leading.” These results suggest that this individual was unwilling to undertake the obligations inherent in their role as an anesthetist; they failed to provide sufficient information to others, neglected to provide feedback, and did not recognize or strive for contentment among their supervisees. Data from the MLQ revealed that this individual typically refrained from engaging in vital matters, was unavailable when needed, evaded decision-making, and procrastinated when asked to respond to pressing inquiries.

Relationship between leadership styles and resilience

**Figure 2:**
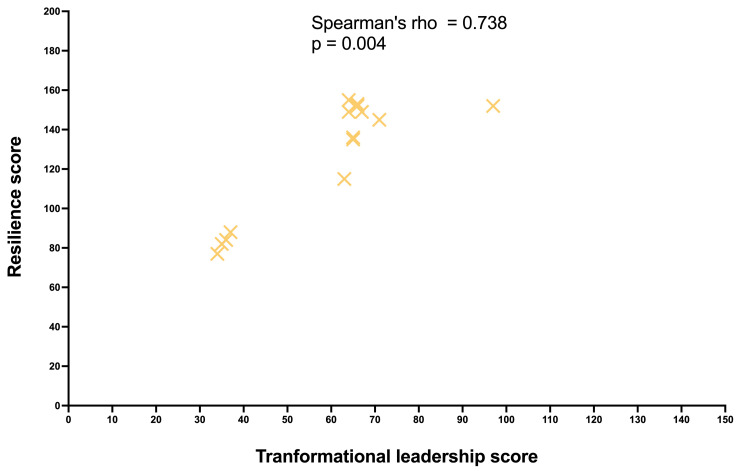
Spearman’s rank correlation assessing the relationship between resilience scores and transformational leadership scores The Spearman’s coefficient (rho) indicates the strength and direction of resilience and transformational leadership scores. Each yellow cross represents a single participant.

**Figure 3:**
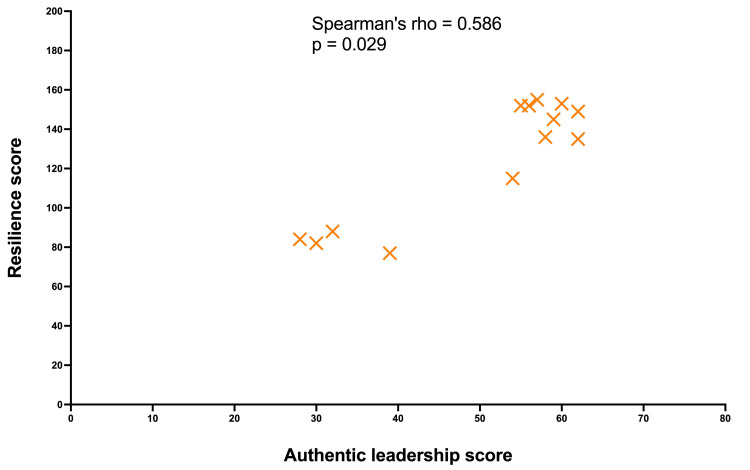
Spearman’s rank correlation assessing the relationship between resilience scores and authentic leadership scores Spearman’s coefficient (rho) indicates the strength and direction of resilience and authentic leadership scores. Each yellow cross represents a single participant.

As shown in Figure 2, Spearman’s rank correlation analysis revealed a strong positive association between resilience and transformational leadership scores (rho = 0.738; 95% CI: 0.326-0.915; p = 0.004). There was a moderate positive association between resilience and authentic leadership scores (rho = 0.586, 95% CI: 0.068-0.88; p = 0.029) (Figure 3). These statistically significant results indicate a positive relationship between higher levels of resilience and stronger transformational and authentic leadership qualities in our study population.

There was no significant association observed between the number of years that participants were in leadership roles and resilience scores (rho = 0.238, 95% CI: -0.350 to 0.692; p = 0.409). Similarly, there was no significant association observed between age and the following variables: authentic leadership scores (rho = 0.256, 95% CI: -0.334 to 0.701; p = 0.374), transformational leadership scores (rho = 0.222, 95% CI: -0.365 to 0.683; p = 0.441), transactional leadership scores (rho = -0.146, 95% CI: -0.638 to 0.431; p = 0.614), and passive-avoidant leadership scores (rho = -0.064, 95% CI: -0.586 to 0.497; p = 0.828).

**Figure 4:**
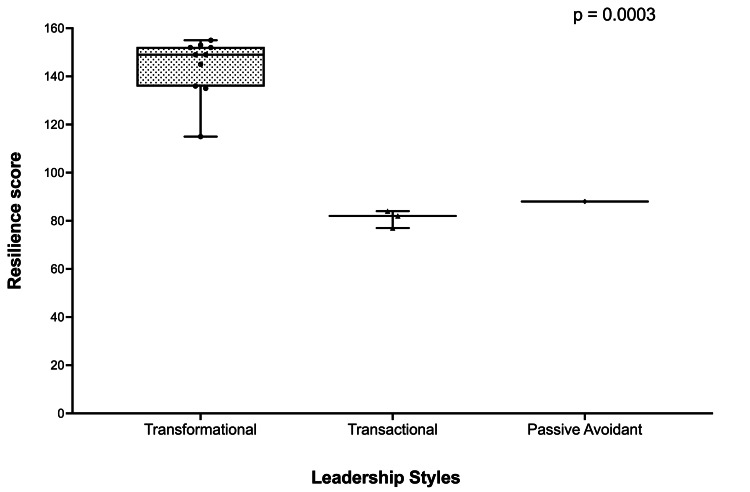
Box and whisker plots showing resilience scores and leadership styles Data presented as median and 25th and 75th quartiles.

As shown in Figure 4, anesthetists with higher authentic leadership scores were more likely to report greater resilience. These findings revealed that these anesthetists as a group were more inclined to foster confidence by transparently disclosing facts and expressing genuine thoughts and emotions. They also reported an inclination to support the importance of individuals, create strong connections, and perceive their authentic selves, encompassing both favorable and unfavorable traits. They also reported a tendency to speak openly about their genuine intentions and desires and to express themselves with the utmost precision.

Anesthetists with higher ALQ scores exhibited strong moral viewpoints that provided them with the capacity to internalize and self-manage. In other words, self-regulation was driven by intrinsic moral principles and ideals rather than extrinsic standards, such as those imposed by one's social circle, healthcare institutions, or the prevailing society. Ultimately, these anesthetists reported a greater equilibrium in their abilities to conduct objective analyses of all pertinent information and actively seek perspectives that question their strongly held beliefs.

Leadership effort, effectiveness, and satisfaction

**Figure 5:**
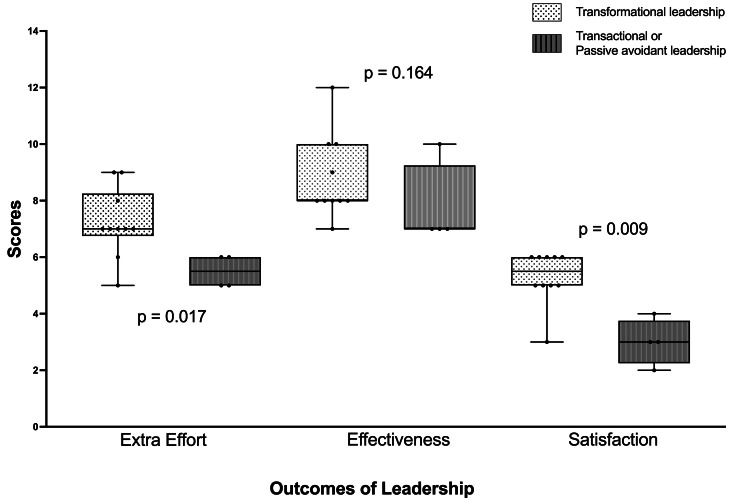
Box and whisker plots showing leadership behavior scores and leadership styles The box shows the median and 25th and 75th quartiles. The whiskers represent the minimum and maximum values. Each dot represents a single participant.

Anesthetists with transformational leadership qualities had significantly higher scores in categories that reflected extra effort exerted and overall role satisfaction (see Figure 5). Specifically, these leaders demonstrated increased levels of motivation, engagement, and initiative among their teams, which translated into consistently higher performance outcomes. The data suggest that transformational leadership traits, such as inspiring and motivating others, fostering an environment of trust, and providing individualized support, were directly associated with enhanced satisfaction. The perception of anesthetists’ effectiveness in their roles did not differ significantly between transformation leaders and transactional or passive/avoidant leaders.

## Discussion

Key findings

To the best of our knowledge, this is the first study to explore the association between anesthetists’ leadership styles and their resilience. Our results revealed that more than two-thirds of anesthetists in leadership roles reported traits consistent with both transformational and authentic leadership. Furthermore, these findings indicate that transformational and authentic leadership styles correlated significantly with measures of resilience.

These findings also indicate that 20% of the anesthetists in leadership positions reported behaviors more consistent with transactional or passive-avoidant leadership. The results of this study reveal that anesthetists who reported these behaviors also reported significantly lower levels of resilience. As a group, these individuals reported putting less effort into their roles, lower levels of effectiveness, and lower levels of job satisfaction compared to those who scored higher for transformational leadership styles.

These findings align with those reported in previous studies and indicate that leadership actions exhibit a strong correlation with job engagement [25,26] and that resilience is a crucial attribute among those hoping to achieve favorable professional outcomes [27]. These findings emphasize that leaders must develop and safeguard their personal and professional resilience to remain effective in demanding and high-pressure healthcare settings and operate within supportive and nurturing healthcare cultures that promote and value resilience [28].

Factors contributing to leadership styles

Many large university hospitals are traditionally considered to have conservative organizational cultures that value stability, adherence to established procedures, and hierarchical structures, all of which may promote transactional leadership. The practice of academic medicine at large university teaching hospitals also tends to be highly regulated. In such settings, transactional leadership may be encouraged as it ensures compliance with standards and processes [29]. Similarly, because large university hospitals tend to consist of multiple layers of management, transactional leadership may be perceived as more practical for maintaining control and consistency. Hospitals driven by short-term goals and metrics may favor transactional leaders who can provide immediate results rather than transformational leaders with a more long-term vision. Similarly, settings with little to no trust between leaders and employees may foster transactional leadership due to a reliance on explicit rules and rewards for compliance.

In contrast, healthcare systems that foster innovation, empower teams, and inspire a shared vision that transcends standard compliance are more likely to encourage a culture of transformational leadership [30]. By prioritizing long-term impact, research excellence, and patient-centered care, hospitals are more likely to develop leaders who inspire change, drive strategic growth, and cultivate a culture of continuous improvement.

Only three out of 14 (21.4%) anesthetists in leadership positions in our study cohort had formal leadership training qualifications. All three displayed transformational leadership traits. In the absence of an adequate leadership training and development program, physicians may default to a transactional leadership style. Moreover, organizations that resist change may discourage transformational leadership that challenges the status quo of traditional and conservative cultures.

Strengths and limitations 

To the best of our knowledge, this is the first study to explore the association between anesthetists’ leadership styles and their resilience. The use of validated standardized questionnaires ensured consistent data collection and analysis, facilitating comparison and generalization. Despite the relatively small leadership group, responses were anonymous, which facilitated more candid and honest responses to sensitive questions. By systematically analyzing the behaviors and resilience strategies of clinical leaders, our findings underscore the importance of fostering resilience as a core component of leadership training, enabling hospitals to cultivate leaders who can navigate the complex, high-stakes environments inherent in healthcare. Finally, our findings can inform the design of leadership programs by emphasizing resilience-building techniques, such as stress management, reflective practice, and team dynamics, ensuring that clinicians in leadership positions are equipped with the skills to lead effectively and sustain high performance. Integrating these insights into leadership development can enhance organizational culture, improve patient care outcomes, and build a more resilient healthcare workforce.

However, the study has several limitations. The present study did not explicitly evaluate the impact of personal qualities, such as self-efficacy, self-awareness, emotional regulation, and drive for improvement. While we acknowledge that these intrinsic characteristics contribute to the cultivation of essential leadership attributes (including effective communication, clinical competence, interpersonal accessibility, and the capacity to serve as an exemplar), it is important to note that our research methodology indirectly addressed these factors. The three validated instruments employed in our study - the Resilience Scale, the Multifactor Leadership Questionnaire, and the Authentic Leadership Questionnaire - contain specific items that assess various dimensions of these personal qualities. Consequently, we posit that many of these individual attributes were inherently captured within the comprehensive framework of our assessment tools, providing an indirect measure of their influence on clinical leadership effectiveness.

We only surveyed anesthetists in leadership roles, and the data may not be generalizable to clinicians in leadership roles, or indeed other anesthetists, in other departments or institutions. Only anesthetist leaders were included in this survey, not the members of staff they lead, who might have very different perspectives on their leadership styles. There could well be a disjuncture between how leaders perceive their leadership and how those being led perceive that leadership. This single-center study, conducted at a university teaching hospital in Australia, may not be generalizable to healthcare settings in other hospitals or countries. Furthermore, the results cannot exclude response bias, i.e., respondents providing inaccurate or socially desirable responses because of perceived hospital expectations.

The study may have been affected by inherent limitations in the questionnaires, such as the absence of strict cutoff scores and/or studied methods to categorize individuals’ favored leadership styles. Instead, these tools reflect a range of behaviors that must be interpreted within their specific and unique contexts. Finally, and most importantly, these questionnaires provide only an initial understanding of the complex issues that contribute to leadership and resilience and may not capture what might be achieved using qualitative research methods. For example, in-depth interviews or focus group research may have provided us with greater insight into other factors contributing to both resilience and leadership styles, such as emotional intelligence, capacity for conflict resolution, and staff burnout.

## Conclusions

In summary, the findings of this study reveal that anesthetists who exhibit transformational and authentic leadership characteristics are likely to be more resilient than those with transactional or passive-avoidant leadership styles. Higher levels of resilience are associated with greater role effectiveness and higher levels of job satisfaction. Future hospital leadership development programs should focus on cultivating transformative leadership abilities through training, workshops, and mentoring programs designed to enhance understanding and the application of transformational leadership practices.

## Appendices

Supplemental File 1: Email invitation

Dear Participant

We would like to invite you to participate in a survey because you are a full-time anaesthetist in our department in a significant leadership role. All anaesthetists in leadership roles in our department have been invited to participate. 

This study will investigate the relationship between leadership styles of anaesthetists in leadership roles and their resilience. 

The email address that will be linked to Qualtrix and used to forward the invitations to participants has been set up by the Austin Health IT Department (anaesthesia.admin@austin.org.au). Neither the Principal Investigator nor the Associate Investigate has access to the account, maintaining blinding and confidentiality. Surveys can be completed online or in paper form. The Department of Anaesthesia Administration team will receive, collate, and deidentify the responses, independent of the investigators. A copy of the deidentified data collection form will be provided to the investigators.

The fundamental objective of this study is to explore the relationship between leadership styles and resilience, not the leadership style of individual anaesthetists and their individual resilience.

The survey is voluntary and will take ~30 minutes to complete. 

All de-identified information collected as part of this survey will be stored in Qualtrics, on the secure Department of Anaesthesia Servers. Your information collected as part of this project can only be accessed by named investigators. By taking part, you will help the researchers understand more about the relationship between leadership styles and resilience. Based on the findings, recommendations may be made to the hospital board to design, develop, implement, and then evaluate leadership and resilience programmes to address and support current and emerging clinicians in leadership roles.

Further information is provided on the attached participant Information Sheet. If you require any further information, please do not hesitate to contact me on my work mobile or via Austin Switch (03 9496 5000)

Attachments: Participant Information Form v1, 26th June 2026

Link to the qualtrics survey from email address anaesthesia.admin@austin.org.au

Supplemental File 2: Participant information form

You have been invited to take part because you are a full-time anaesthetist in our department in a significant leadership role. All anaesthetists in leadership roles in our department have been invited to participate. Anaesthetists invited to participate include: 

1. Head of anaesthesia at the Surgical Centre

2. Deputy directors of anaesthesia 

3. Head of cardiac anaesthesia

4. Deputy head of cardiac anaesthesia

5. Head of liver transplantation anaesthesia

6. Head of anaesthesia for perioperative medicine

7. Deputy head of anaesthesia for perioperative medicine

8. Head of acute pain medicine

9. Head of chronic pain medicine

10. Head of anaesthesia research

11. Supervisors of training

This study will investigate the relationship between leadership styles of anaesthetists in leadership roles and their resilience. 

Our department has recently been through significant challenges, especially navigating the coronavirus disease (COVID-19) caused by the SARS-CoV-2 virus. Your role in helping to navigate many of these challenges both within our department and across the broader hospital network has been invaluable and critical. 

You will be sent an email inviting you to participate in this study. The email address that will be used to forward the invitations to participants has been set up by the Austin Health IT department (anaesthesia.admin@austin.org.au). Neither the Principal Investigator nor the Associate Investigate has access to the account, maintaining blinding and confidentiality. Surveys can be completed online or in paper form. The Department of Anaesthesia Administration team will receive, collate, and deidentify the responses, independent of the investigators. A copy of the deidentified data collection form will be provided to the investigators. 

The investigators will not be able to identify individual anaesthetists from their responses. The fundamental objective of this study is to explore the relationship between leadership styles and resilience, not the leadership style of individual anaesthetists and their individual resilience.

Taking part is up to you.

You get to decide whether you want to continue to take part. You can say no if you want to.

Your decision won’t affect your relationship with the Department of Anaesthesia or with Austin Health. 

You can change your mind at any time.

If you do take part, you can stop at any time. If you want to stop, you do not have to tell us the reason. If you decide to stop taking part, none of the de-identified data that has been collected will be used. We will not keep the information we have already collected.

If you are happy to take part in the study, you will be asked to complete 3 validated questionnaires. If you choose to complete the data collection and submit it for analysis, this will be evidence of your implied consent to participate in the study. You can fill these questions out on paper or if you prefer you can complete them online. There will be no way that you will be able to be identified. Once you have completed the questionnaires, there will be no follow-up in any way.

In total, the questionnaires will take approximately 30 minutes to complete.

What are the questionnaires?

Tell me more about each questionnaire.

Multifactor Leadership Questionnaire (MLQ)

This MLQ is a psychological inventory consisting of 36 items pertaining to leadership styles and 9 items pertaining to leadership outcomes. The MLQ was constructed with the goal to assess a full range of leadership styles. The MLQ is composed of 9 scales that measure three leadership styles: transformational leadership, transactional leadership, and passive/avoidant behaviour. 

This will take ~15 minutes to complete.

Authentic Leadership Questionnaire (ALQ)

This questionnaire assesses the self-awareness, transparency, ethics/morality, and processing ability of leaders. The ALQ is a 16-item instrument that measures an individual's authentic leadership behaviours. 

This will take ~5 minutes to complete.

The Resilience Scale Questionnaire

The Resilience Scale is a validated 25-item questionnaire that measures resiliency in the work environment. The questionnaire identifies five essential characteristics of resilience, namely Self-reliance, Purpose, Equanimity, Perseverance, and Authenticity). 

This will take ~10 minutes to complete

By taking part, you will help the researchers understand more about the relationship between leadership styles and resilience. Based on the findings, recommendations may be made to the hospital board to design, develop, implement, and then evaluate leadership and resilience programmes to address and support current and emerging clinicians in leadership roles.

The only inconvenience is that the survey requires 30 minutes of time to complete. To minimise the risk of inconvenience, you can complete the survey at a time that is convenient to you in paper form or online.

Any answers to the questions are completely de-identifiable. All data will be summarised, in a deidentified format and will be completely anonymised for the purpose of this study. Given that all data will be de-identified, it will not be possible to have access to your own results nor the results of anyone else participating. A summary statement outlining the findings will be provided to everyone who participates. We will keep this de-identified information for 7 years. After this, we will destroy it.

The research will be published in a journal and may be presented to the Austin Health board.

This project is being run only at Austin Health. This project is being funded by the Department of Anaesthesia.

The Austin Health Human Research Ethics Committee has approved this project. This committee makes sure that this project meets Australian ethical standards for research that involves people. 

Thank you for taking the time to read this information about our project. You can contact a member of the project team at any time to ask questions.

Supplemental File 3: Questionnaires

**Table 2:** The Multifactor Leadership Questionnaire The authors have acquired a license to administer the Authentic Leadership Questionnaire and Multifactor Leadership Questionnaire from MindGarden. Source: Ref [11]

MLQ - Multifactor Leadership Questionnaire^TM ^Leader Form (Sx-Short)				
For use by Laurence Weinberg only. Received from Mind Garden, Inc. on November 29, 2023.				
This questionnaire is to describe your leadership style as you perceive it. Please answer all items on this answer sheet. If an item is irrelevant, or if you are unsure or do not know the answer, leave the answer blank. Forty-five descriptive statements are listed on the following pages. Judge how frequently each statement fits you. The word “others” may mean your peers, clients, direct reports, supervisors, and/or all of these individuals.				
Use the following rating scale: 0 (Not at all); 1 (Once in a while); 2 (Sometimes); 3 (Fairly often); 4 (Frequently, if not always)				
I provide others with assistance in exchange for their efforts	0	2	3	4
I re-examine critical assumptions to question whether they are appropriate	0	2	3	4
I fail to interfere until problems become serious	0	2	3	4
I focus attention on irregularities, mistakes, exceptions, and deviations from standards	0	2	3	4
I avoid getting involved when important issues arise	0	2	3	4
I talk about my most important values and beliefs	0	2	3	4
I am absent when needed	0	2	3	4
I seek differing perspectives when solving problems	0	2	3	4
I talk optimistically about the future	0	2	3	4
I instill pride in others for being associated with me	0	2	3	4
I discuss in specific terms who is responsible for achieving performance targets	0	2	3	4
I wait for things to go wrong before taking action	0	2	3	4
I talk enthusiastically about what needs to be accomplished	0	2	3	4
I specify the importance of having a strong sense of purpose	0	2	3	4
I spend time teaching and coaching	0	2	3	4
I make clear what one can expect to receive when performance goals are achieved	0	2	3	4
I show that I am a firm believer in “If it ain’t broke, don't fix it”	0	2	3	4
I go beyond self-interest for the good of the group	0	2	3	4
I treat others as individuals rather than just as a member of a group	0	2	3	4
I demonstrate that problems must become chronic before I take action	0	2	3	4
I act in ways that build others' respect for me	0	2	3	4
I concentrate my full attention on dealing with mistakes, complaints, and failures	0	2	3	4
I consider the moral and ethical consequences of decisions	0	2	3	4
I keep track of all mistakes	0	2	3	4
I display a sense of power and confidence	0	2	3	4
I articulate a compelling vision of the future	0	2	3	4
I direct my attention toward failures to meet standards	0	2	3	4
I avoid making decisions	0	2	3	4
I consider an individual as having different needs, abilities: and aspirations from others	0	2	3	4
I get others to look at problems from many different angles	0	2	3	4
I help others to develop their strengths	0	2	3	4
I suggest new ways of looking at bow to complete assignments	0	2	3	4
I delay responding to urgent questions	0	2	3	4
I emphasize the importance of having a collective sense of mission	0	2	3	4
I express satisfaction when others meet expectations	0	2	3	4
I express confidence that goals will be achieved	0	2	3	4
I am effective in meeting others' job-related needs	0	2	3	4
I use methods of leadership that are satisfying	0	2	3	4
I get others to do more than they expected to do	0	2	3	4
I am effective in representing others to higher authority	0	2	3	4
I work with others in a satisfactory way	0	2	3	4
I heighten others• desire to succeed	0	2	3	4
I am effective.in meeting organizational requirements	0	2	3	4
I increase others' willingness to try harder	0	2	3	4
I lead a group that is effective	0	2	3	4
©1995 Bruce Avolio and Bernard Bass. All rights reserved in all media. Published by Mind Garden. Inc. www.mindgarden.com				

**Table 3:** The Authentic Leadership Questionnaire The authors have acquired a license to administer the Authentic Leadership Questionnaire and Multifactor Leadership Questionnaire from MindGarden. Source: Ref [13]

Authentic Leadership Questionnaire (ALQ Version 1.0 Self)				
For use by Laurence Weinberg only. Received from Mind Garden, Inc. on December 24, 2023.				
Instructions: The following survey items refer to your leadership style, as you perceive it. Please judge how frequently each statement fits your leadership style using the following scale: 0 (Not at all); 1 (Once in a while); 2 (Sometimes); 3 (Fairly often); 4 (Frequently, if not always)				
As a leader, I				
say exactly what I mean	0	2	3	4
admit mistakes when they are made	0	2	3	4
encourage everyone to speak their mind	0	2	3	4
tell you the hard truth	0	2	3	4
display emotions exactly in line with feelings	0	2	3	4
demonstrate beliefs that are consistent with actions	0	2	3	4
make decisions based on my core values	0	2	3	4
ask you to take positions that support your core values	0	2	3	4
make difficult decisions based on high standards of ethical conduct	0	2	3	4
solicit views that challenge my deeply held positions	0	2	3	4
analyze relevant data before coming to a decision	0	2	3	4
listen carefully to different points of view before coming to conclusions	0	2	3	4
seek feedback to improve interactions with others	0	2	3	4
accurately describe how others view my capabilities	0	2	3	4
know when it is time to reevaluate my position on important issues	0	2	3	4
show I understand how specific actions impact others	0	2	3	4
©1995 Bruce Avolio and Bernard Bass. All rights reserved in all media. Published by Mind Garden. Inc. www.mindgarden.com				

**Table 4:** The Resilience Questionnaire The authors have obtained permission from Professor Gail M. Wagnild, the owner and CEO of the Resilience Centre, to utilize the Resilience Questionnaire. Source: Ref [18]

RESILIENCE SCALE^TM^						
Date:						
Please read each statement and circle the number to the right of each statement that best indicates your feelings about the statement. Respond to all statements.						
Circle the number in the appropriate column.	Strongly Disagree					Strongly Agree
1. When I make plans, I follow through with them.	1	2	3	4	5	6
2. I usually manage one way or another.	1	2	3	4	5	6
3. I am able to depend on myself more than anyone else.	1	2	3	4	5	6
4. Keeping interested in things is important to me.	1	2	3	4	5	6
5. I can be on my own if l have to.	1	2	3	4	5	6
6. I feel proud that I have accomplished things in life.	1	2	3	4	5	6
7. I usually take things in stride.	1	2	3	4	5	6
8. I am friends with myself.	1	2	3	4	5	6
9. I feel that I can handle many things at a time.	1	2	3	4	5	6
10. I am determined.	1	2	3	4	5	6
11. I seldom wonder what the point of it all is.	1	2	3	4	5	6
12. I take things one day at a time.	1	2	3	4	5	6
13. I can get through difficult times because I've experienced difficulty before.	1	2	3	4	5	6
14. I have self-discipline.	1	2	3	4	5	6
15. I keep interested in things.	1	2	3	4	5	6
16. I can usually find something to laugh about.	1	2	3	4	5	6
17. My belief in myself gets me through hard times.	1	2	3	4	5	6
18. In an emergency, I'm someone people can generally rely on.	1	2	3	4	5	6
19. I can usually look at a situation in a number of ways.	1	2	3	4	5	6
20. Sometimes I make myself do things whether I want to or not.	1	2	3	4	5	6
21. My life has meaning.	1	2	3	4	5	6
22. I do not dwell on things that I can't do anything about.	1	2	3	4	5	6
23. When I'm in a difficult situation, I can usually find my way out of it.	1	2	3	4	5	6
24. I have enough energy to do what I have to do.	1	2	3	4	5	6
25. It's okay if there are people who don't like me.	1	2	3	4	5	6
©1993 Gail M. Wagnild and Heather M. Young. Used by permission. All rights reserved. “The Resilience Scale” is an international trademark of Gail M. Wagnild & Heather M. Young, 1993.						
